# Problems with Peer Review Shine a Light on Gaps in Scientific Training

**DOI:** 10.1128/mbio.03183-22

**Published:** 2023-04-13

**Authors:** Diana M. Proctor, Nsa Dada, Anna Serquiña, Julia L. E. Willett

**Affiliations:** a National Institutes of Health, National Human Genome Research Institute, Translational and Functional Genomics Branch, Microbial Genomics Section, Bethesda, Maryland, USA; b School of Life Sciences, Arizona State University, Tempe, Arizona, USA; c National Institutes of Health, National Cancer Institute, Center for Cancer Research, Bethesda, Maryland, USA; d University of Minnesota Medical School, Minneapolis, Minnesota, USA

**Keywords:** education, peer review, training

## EDITORIAL

The term “peer review” often brings to mind the process by which scientific journals solicit experts to evaluate manuscripts prior to their publication. Scientists have debated the utility and value of peer review since its modern deployment in the 1970s (1). Problems with peer review and concerns about its role in scientific gatekeeping have led some to propose, in scientific manuscripts (2, 3) and in media articles (4), eliminating peer review altogether. One major concern is that peer review has done little to identify failures of scientific rigor (e.g., improper statistics, missing controls, data fabrication, or manipulation, detection of bias) (5, 6). Another issue is that it has revealed institutional, geographic, racial, and gender bias (7, 8). To address these problems, recent innovations in peer review include blinding, double blinding, open peer review, and “community peer review” in the form of open, public feedback on preprint servers and on other scientific forums. Taking a step back, we wonder whether current problems with peer review point to gaps in scientific training. Whether or not scientists continue to perform peer review as a service to scientific journals, we must still be able to identify major errors in scientific manuscripts, but current evidence suggests that we cannot. While post-publication review by the scientific community can eventually right publishing wrongs or present corrections to flawed studies, this can be a lengthy process that consumes time, resources, and funding (9). Rather than eliminate peer review, we propose that we use the process of peer review as an instrument to train scientists on how to evaluate science critically, fairly, and civilly through the development of formal peer review training programs. Unfortunately, this kind of training program is not widely implemented at any level of scientific training.

**FIG 1:**
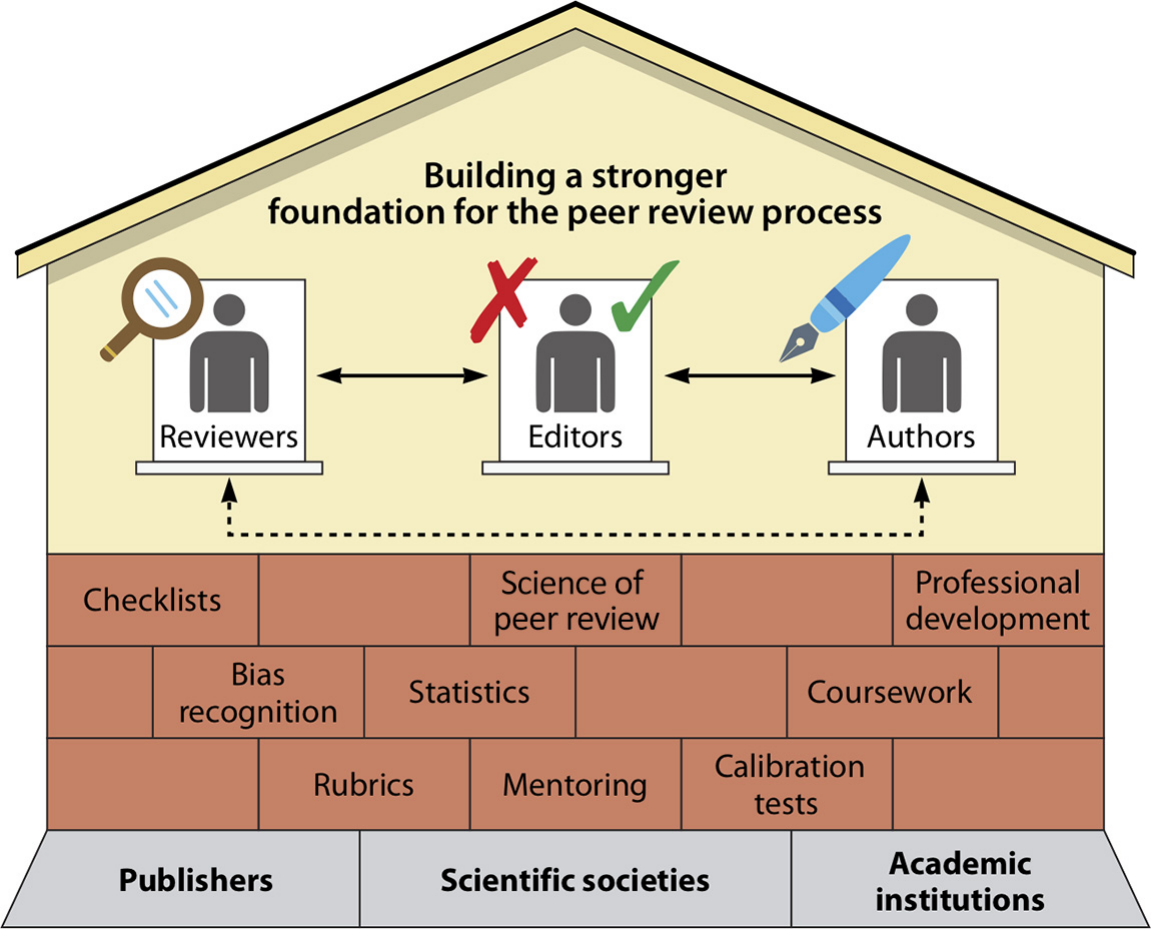
Fortifying the foundation of peer review through structured training programs. The stakeholders in the peer review process are the editors who represent journals, authors, and reviewers. During traditional peer review, authors draft manuscripts (denoted by the blue pen), reviewers examine and critique manuscripts (denoted by the magnifying glass), and editors reject or accept manuscripts (denoted by the red X or green check, respectively) based in part on input from reviewers. Publishers, societies, and academic institutions form the foundation by which peer review can be strengthened through training. Existing training programs are provided by some of these groups. We propose core components of training programs (denoted as bricks, which can be implemented by multiple stakeholders) to strengthen the foundation of peer review through coursework, professional development, structured evaluations, and other elements described in Table 1. The arrows show the two-way relationships between stakeholders and the collaboration necessary to improve peer review. Additionally, community review can also occur directly between authors and reviewers.

What problems with scientific training has peer review illuminated? In our current system, many scientists gain experience with peer review by “learning on the job” when invited to review by an editor or as apprentices contributing to reviews accepted by mentors. The lack of formal training may underlie the expectation gap between what biomedical editors report wanting to see in peer reviews and what referees deliver. Editors – often leading scientists in the field – identify core problems with submitted peer reviews, including unacceptable text or tone, requests for additional experiments outside the scope or peer review guidelines of the journal, and misuse of the confidential notes to editors (2, 10). Additionally, although journal editors currently have the responsibility and power of removing inappropriate comments from peer reviews, receiving scathing reviews is unfortunately a common experience. Uncivil peer review comments, in turn, disproportionately harm early career researchers and scientists from historically excluded and marginalized communities (11). Closing the gap between the goals of peer review and reality may also be hindered by a paucity of objective scoring rubrics for editors to rate review quality. Reviews are frequently evaluated subjectively, yet there is only modest agreement among editorial assessments of peer review quality (12). Perhaps review quality could be increased by structured training of scientists, including editors, on peer review as well as on bias, which has been shown to skew publishing outcomes (8, 13). We propose that such improvement requires coordinated training programs involving all aspects of publishing, including authors, peer reviewers, scientific societies, and publishers (Fig. 1).

The science of peer review suggests a path for developing training programs. Data indicate that a background in statistical or epidemiological analysis is predictive of a higher editorial rating of review quality (14). As a result, focused training in evaluation of study execution and the use of resources, such as scoring rubrics or checklists, is needed to improve evaluation of the statistical rigor of manuscripts (15). Additionally, journals could consider hiring dedicated statistical editors who vet all manuscripts. While previous studies of peer review training programs found that short interventions failed to or only transiently increased subjective ratings of review quality, it’s possible that longer, more structured programs that target a larger set of major issues with peer review will result in sustained improvements (16, 17). Calibrating reviewers on test manuscripts may enable quantitative assessment of our ability to accurately identify issues with methodology or data analysis (including omission of control experiments, improper statistical tests, or missing components, such as sequence deposition information). An introduction to civility and professional norms of communication, including theory, structured examples, and discussion of what constitutes appropriate or inappropriate phrasing, tone, and commentary (18), should also be emphasized in peer review training programs. Based on the literature, we propose a framework for peer review training that includes modules on implicit bias, peer review as a science, ethics, civility, and professional norms of communications, and resources to improve inter-rater reliability (Table 1).

**Table 1:** Proposed components to consider including in peer review training programs

**Implicit bias training and testing.** Data indicate that bias in scientific peer review results in biased publication outcomes (reviewed in [7]) but that awareness of bias can mitigate it. As a result, we recommend teaching about and testing for implicit bias, rather than assuming this training occurs elsewhere (https://plato.stanford.edu/entries/implicit-bias/ [21]). **Didactic instruction on the science of peer review**. As of the date of this writing, over 25,000 articles on “peer review” have been deposited in PubMed. Peer review training programs should introduce scientists to this literature, using data to teach scientists about the history of peer review, common problems in the process, and data-driven solutions to pitfalls. **Instruction on use of community-wide resources on peer review.** COPE, an organization that guides editors, has developed guidelines for peer review and scientific publishing, which should be covered in training programs (https://publicationethics.org/files/ethical-guidelines-peer-reviewers-cope.pdf). In addition, programs should teach individuals to consult journal-specific guidance on peer review prior to accepting any invitation. For instance, guidance differs between ASM journals (https://journals.asm.org/reviewer-guidelines). As a result, scientists need to be taught to consult the specifics of each journal’s guidelines before accepting an invitation to review, so they know they’re agreeing to use journal specific standards, including scope, scoring rubrics/templates, and evaluation of significance or impact during the peer review process. **Instruction on how to evaluate a manuscript.** Students should be taught how to evaluate manuscripts for author bias, statistical rigor, adequacy of controls, reproducibility of methods, transparency of data, adequacy of reference lists, how well data support conclusions, and adequacy of discussion of study limitations. Checklists serve as a safeguard against bias and may also increase transparency in the peer review process (15). Programs should instruct reviewers to use checklists to standardize evaluation of manuscripts. **Evaluation using calibration exercises.** Course instructors should develop a set of peer review training manuscripts that can be used to evaluate whether scientists can adequately detect common errors in manuscripts (12). Inter-rater reliability scores can be calculated to determine what scientific errors scientists commonly fail to detect, and course content or checklists can be adapted to provide instruction to address deficiencies. **Instruction on how to write a peer review.** Instruction should cover content and style of peer reviews. Peer reviews are commonly comprised of (i) an introductory paragraph which serves to summarize the major findings of a manuscript and its place in the scientific literature (i.e., significance), (ii) major comments which outline revision requests that address deficiencies in scientific rigor that must be addressed prior to publication, and (iii) minor comments which outline ways the authors could revise the manuscript to increase its readability. Training programs should use examples (either public peer reviews from preprint servers or examples generated by the instructors) to teach scientists what comments belong in major and minor comments, and whether introductory paragraphs are sufficiently written. In terms of style, instruction should focus on the appropriate professional tone to use in peer review, evaluating published examples for context. **Instruction on the ethics of scientific publishing.** This module should cover the peer reviewer’s role in safeguarding against (i) scientific misconduct in papers, (ii) biases commonly seen in manuscripts, and (iii) ethical responsibilities of peer reviewers. **Instruction on communication with editors.** The confidential note to editors is commonly mis-used or under-used by reviewers. Peer reviewers should be taught (i) how to use this box appropriately as well as other bounds surrounding communications with editors, (ii) when to decline a review, and (iii) how to report alleged scientific misconduct, including plagiarism, and image manipulation, etc. **Instruction of civility and professional communication.** Students should be taught about civility, inter-generational views of civility, and the norms of professional communications in science (18). **Long-term, structured mentoring and feedback.** Currently, many scientists are trained to conduct peer review by their mentors, using the apprenticeship model of training. Peer group training, used by the mBio JEB, is another model. Peer review training programs should consider guiding scientists through the process of evaluating several manuscripts, providing feedback on review quality and process, given that current evidence indicates that short training programs provide minimal long-term improvements in peer review.

Who should be responsible for managing peer review training? Some publishers, like Nature Publishing Group, already offer online training courses (https://masterclasses.nature.com/online-course-on-peer-review/16507836), but training is not mandated for manuscript reviewers, and training in study design, bias, and civility aren’t included. Some institutions offer courses on peer review through their center for teaching and learning, but courses are typically not part of required curricula. Scientific societies - like the American Society for Microbiology (ASM) - could integrate training into existing publishing mechanisms. Many societies, including ASM, offer journal-specific reviewer guidelines or educational webinars. In 2021, ASM launched the inaugural mBio Junior Editorial Board (JEB) to train early career researchers on various aspects of scientific publishing. In our first year, JEB members participated in a monthly seminar series on scientific publishing (including peer review) and conducted 2 mentored peer reviews, which were evaluated by mBio editors. In our second year, JEB members review manuscripts submitted to mBio while getting editorial feedback on review quality, process, and scope. Future directions involve scaling JEB across all ASM journals and eventually creating a comprehensive, ASM-wide program. This editorial was largely motivated by our experience with JEB - and our variable experiences with peer training groups - which led us to consider ways to improve the ASM program, the purpose of peer review, and the components that ought to be included in rigorous peer review training programs.

What are the arguments against peer review training programs? From the journal’s perspective, implementing rigorous training programs must be balanced with the possibility that requiring training may decrease the pool of peer reviewers, potentially increasing the time manuscripts spend in review. Experienced peer reviewers may be unwilling to commit additional time and energy to training as a result of time constraints, lack of compensation, lack of awareness of current problems with peer review outcomes, or resistance to modifying personal reviewing strategies. Additionally, if individual societies, journals, and publishers all create their own peer review training program, each with its own best practices and guidelines, we risk fragmenting peer review beyond a reasonable scope.

We propose integrating structured peer review training into coursework at the undergraduate and graduate levels. Undergraduate research fellowships and programs are widespread, and research is often a requirement to earn degree honors or distinction. Undergraduates are frequently required to read scientific articles in their courses, so instruction on peer review can provide a tangible example of the practical importance of their coursework. Publishing peer-reviewed manuscripts is a key component of graduate research and a requirement for earning a PhD in many departments, making coursework an ideal forum for teaching about the process of peer review. In addition to offering *de novo* peer review courses, training can be folded into existing coursework already designed to teach scientific literacy. Most PhD programs train students in scientific literacy through qualifying exams and classes, which are typically designed to prepare students to write an NIH-style grant or fellowship (even though the majority of PhD students will obtain jobs where they will not write grants, but may participate in peer review). Additionally, journal clubs serve as a platform for teaching students to evaluate and present data from manuscripts and can be adapted to introduce best practices for peer review. Incorporating peer review into scientific education may also help combat inappropriate tone and content of reviews (such as personal attacks) by providing a formal venue to teach students and early career researchers norms of professional communications and how to collaborate with editors to resolve inappropriate reviews.

Implementation of peer review training programs by academic institutions requires buy-in from stakeholders at multiple levels. Using frameworks developed in studies on coordination and collaboration (19, 20), we identified high-level buy-in targets include funding, rewards, and external pressure. Peer review education could be tied to training grants, especially those from NIH that already mandate Responsible Conduct of Research training. Rewards systems, such as rewarding student/mentor pairs who excel at reviewing manuscripts or teaching about peer review, could be implemented by individual departments and universities, by publishers, and by scientific societies. External stakeholders for post-undergraduate and post-graduate careers (such as graduate admission programs or postdoctoral fellowship evaluation committees) can require coursework in peer review as a pre-requisite, similar to requiring courses in biology, chemistry, and statistics.

Peer review, whether as a service to journals, in the lab, or in the classroom, can be an instrument to teach us how to think rigorously about science. Current evidence suggests major problems with peer review that shine a light on deficiencies in current educational programs. Peer review training, whether implemented by scientific societies, publishing groups, or as part of graduate education, need not operate in silos. As early career scientists, we advocate for each of these stakeholders to collaborate in fortifying the peer review process to reduce bias in publication outcomes and improve the integrity of the foundation upon which the future of science is built.
